# Leonurine, a potential drug for the treatment of cardiovascular system and central nervous system diseases

**DOI:** 10.1002/brb3.1995

**Published:** 2020-12-10

**Authors:** Lu Huang, Ding‐Qiao Xu, Yan‐Yan Chen, Shi‐Jun Yue, Yu‐Ping Tang

**Affiliations:** ^1^ Key Laboratory of Shaanxi Administration of Traditional Chinese Medicine for TCM Compatibility State Key Laboratory of Research & Development of Characteristic Qin Medicine Resources (Cultivation) Shaanxi Key Laboratory of Chinese Medicine Fundamentals and New Drugs Research Shaanxi Collaborative Innovation Center of Chinese Medicinal Resources Industrialization Shaanxi University of Chinese Medicine Xi’an China

**Keywords:** cardiovascular system, central nervous system, leonurine, *Leonurus*, structure–activity

## Abstract

*Leonurus japonicus* Houtt., a traditional Chinese herbal medicine, is often used as a gynecological medicine with the effect of promoting blood circulation, regulating menstruation, clearing heat, and detoxificating. As the most important alkaloid in *L. japonicus*, leonurine has a wide range of biological activities, such as antioxidation, anti‐inflammation, and anti‐apoptosis. Cardiovascular system and central nervous system diseases are arrogant killers that threaten human lives and health around the world, but many drugs for treating them have certain side effects. This paper reviews the potential therapeutic effects of leonurine on cardiovascular system and central nervous system diseases, summarizes the previous research progress, and focuses on its therapeutic effect in various diseases. Although leonurine plays a prominent role in the treatment of cardiovascular system and central nervous system diseases, there are still some shortages, such as low bioavailability, weak transmembrane ability, and poor fat solubility. Therefore, the structure modification of leonurine may solve these problems and provide reference value for the development of new drugs. At present, leonurine is in clinical trial, and it is hoped that our summary will help to provide guidance for its future research on the basic science and clinical application.

## Graphical abstract

This article reviews the pharmacological effects of a hot potential drug leonurine on cardiovascular and brain diseases. Its structure modification may be an effective way to reduce side effects, strengthen efficacy, and improve bioavailability. We believe that in the near future, leonurine is very likely to become one of the new drugs following the results of artemisinin, which will benefit all human beings.

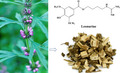

## 1 INTRODUCTION

**Figure 1:**
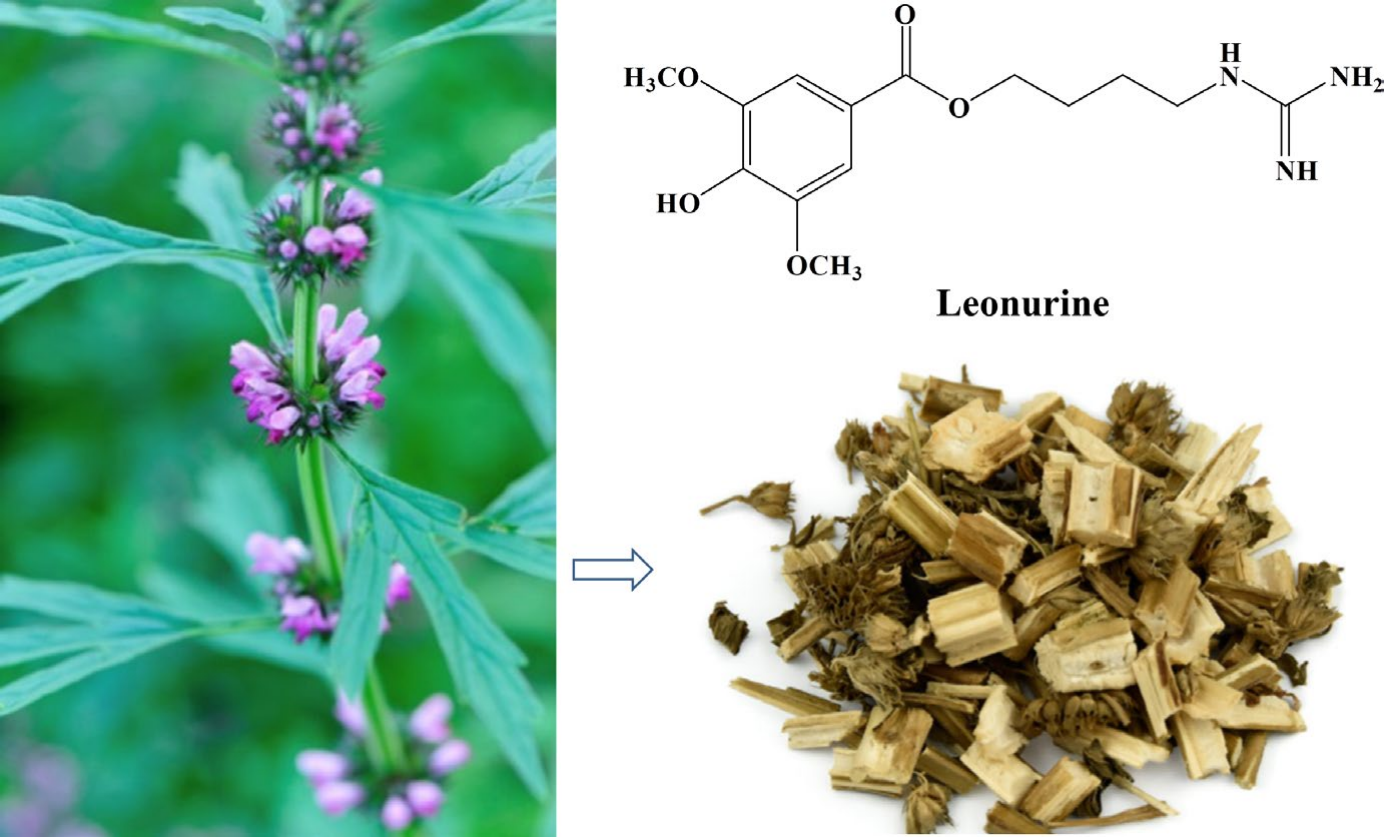
The photograph of *Leonurus japonicus* Houtt. and the structure of leonurine

*Leonurus japonicus* Houtt., also called “Yi‐Mu‐Cao” in Chinese, is often widely used as a gynecological drug to promote blood circulation and regulate menstruation recorded in *Chinese Pharmacopoeia*. More than two hundred chemical compounds have been separated, including alkaloids, terpenoids, and flavonoids. Among these, alkaloids are the most important bio‐active compounds, especially leonurine, which was also known as SCM‐198 and was reported to be the most crucial constituent of *Leonurus japonicas* (Figure 1) (Liu, Zhang, et al., 2012). It has been demonstrated that leonurine has extensive range of biological activities, including anti‐inflammation (Song et al., 2015), antioxidant activity (Sun et al., 2005), anti‐platelet aggregation activity (Zhou et al., 1996), excited uterine activity (Li et al., 2013), as well as anti‐tumor activity (Mao et al., 2015), cardiovascular protective effects (Zhu et al., 2018), and brain protection (Liu, Zhang, et al., 2012). However, the content of leonurine in *L. japonicus* is only 0.02%–0.12%, which makes it difficult to extract and separate the compound, and it is also not easy to apply leonurine in clinic (Liu, Pan, et al., 2013; Xiong & Peng, 2016). Despite having these shortcomings, it still cannot prevent many researchers from studying and exploring it, and its wide bio‐activities are attracting more and more attention around the world.

Traditional Chinese medicine (TCM) believes that the heart is the core of the human body, while contemporary medicine regards that the brain is the core. However, studies have found that there is a close relationship between heart and brain, and there is a mutually reinforcing relationship between cardiovascular system and central nervous system diseases, both of which have become the biggest killers threatening human health (Meloux et al., 2018). Unhealthy lifestyle and irregular eating habits are the potential causes of these diseases. The main cardiovascular system diseases include atherosclerosis, myocardial infarction and heart disease. Central nervous system diseases include cerebrovascular diseases such as ischemic stroke, traumatic brain injury disease, neurodegenerative disorder such as Alzheimer′s disease and Parkinson′s disease, and mental illness such as depression (Prabhakaran et al., 2018). These diseases were ordinary treated with chemical medicines, but their side effects cannot be ignored. Early studies showed that leonurine had a contractile and diastolic effect on blood vessels, which suggested that leonurine might have a potential therapeutic effect on cardiovascular and cerebrovascular diseases (Chen & Kwan, 2001). Some studies in recent decades have found that leonurine not only treat atherosclerosis induced by hypercholesterolemia, but also significantly reduced the infarct area of cerebral cortex caused by cerebral ischemia and improved the symptoms of neurological injury (Qi et al., 2010). So, the potential therapeutic effects of leonurine on some cardiovascular system and central nervous system diseases are mainly discussed in the present review, and its structural features and structure–activity relationship are also touched upon.

## 2 POTENTIAL CLINICAL APPLICATION OF LEONURINE

Two decades ago, leonurine was report to has the effect of strengthening uterus and diuresis, and then some scholars discovered its anti‐inflammation, anti‐apoptosis and antioxidation, which proved that leonurine not only has a good treatment for cardiovascular system diseases, but also has potential effects on central nervous system diseases such as stroke and brain injury (Liu et al., 2010; Loh et al., 2010). Although there are many drugs on the market for these diseases, most of the drugs are expensive with not good treatment action, and their frequent use would bring a lot of side effects to patients. At the present time, curing cardiovascular system and central nervous system diseases is still a worldwide problem. Many studies have shown that leonurine had a good therapeutic effect, and it is also easy to synthesize it with high yield and good purity in industry (Luo & Gu, 2012). Therefore, leonurine's therapeutic ability for cardiovascular system and central nervous system diseases has become a hot topic, and some scientists even predict that leonurine may be very promising as a new cardioprotective agent (Luo & Gu, 2012).

### 2.1 Treatment of cardiovascular system diseases

**Figure 2:**
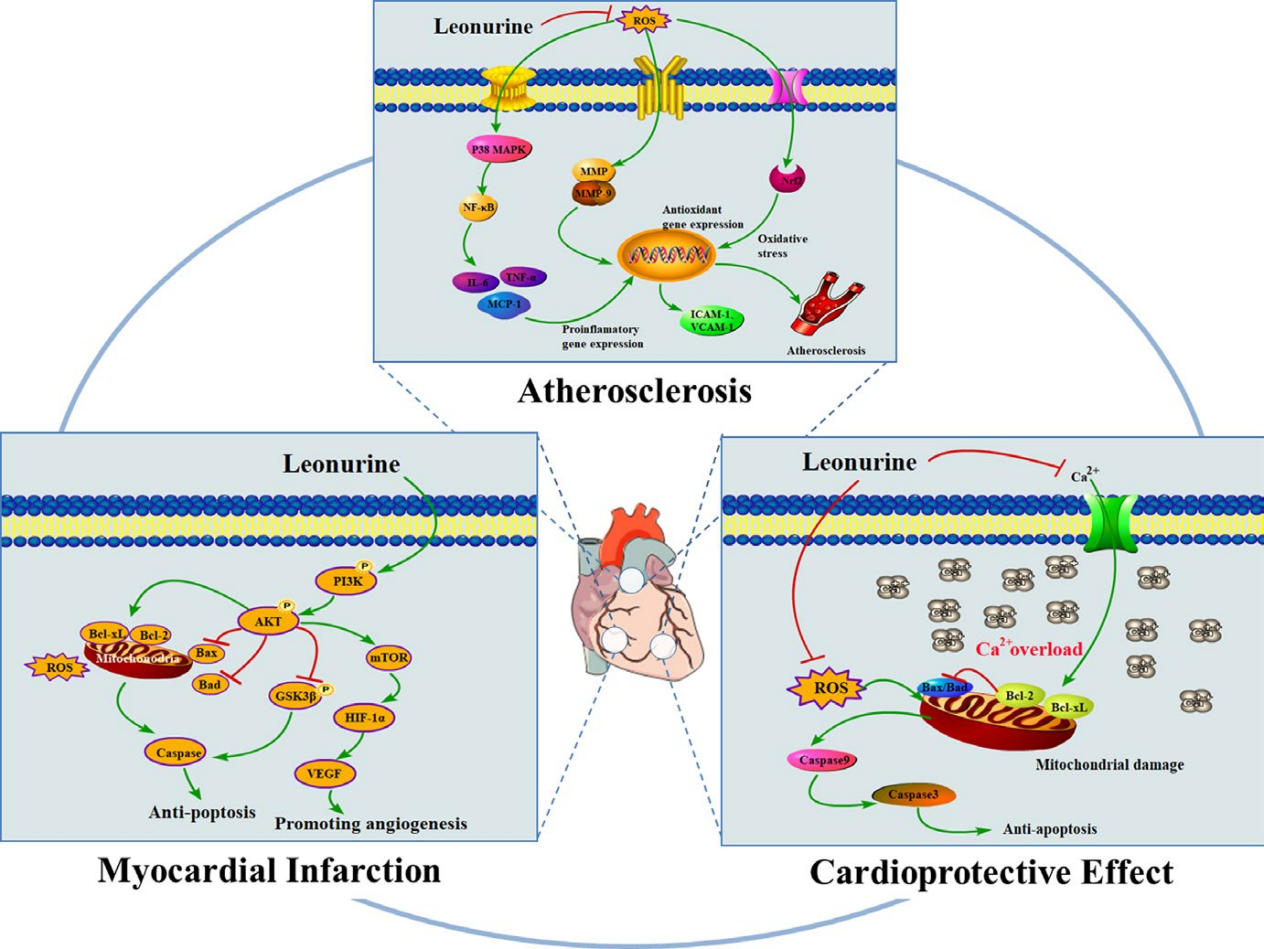
Potential role of leonurine in cardiovascular disease. Leonurine can treat cardiovascular disease by anti‐atherosclerosis, reducing myocardial infarction and cardioprotective effects. It plays a strong protective role through a wide range of mechanisms. In atherosclerosis, leonurine plays an important role in reducing hypercholesterolemia through antioxidant stress and anti‐inflammation, which is of great significance to the treatment of AS. In myocardial infarction, leonurine activated PI3K/Akt signal pathway, down‐regulated the expression of Bax and Fas, and up‐regulated the expression of Bcl‐2 and Bcl‐xL. At the same time, leonurine promoted myocardial contraction and angiogenesis by up‐regulating mTOR/ERK signal pathway. In addition, leonurine also has cardioprotective effects by reducing calcium overload and anti‐apoptosis effects

Long‐term studies have shown that leonurine has a significant therapeutic effect on cardiovascular system diseases, such as atherosclerosis, myocardial infarction and myocardial ischemia. Leonurine can ameliorate cardiovascular diseases and show cardioprotective effect. Therefore, leonurine is entering clinical trials as a new drug for the treatment of cardiovascular diseases. This section reviews the treatment effects of leonurine on some common cardiovascular system diseases (Figure 2).

#### 2.1.1 Treatment of atherosclerosis

Atherosclerosis (AS) is a common cardiovascular disease caused by hyperlipidemia, hypertension, diabetes, smoking, obesity, and other causes (van Rooy & Pretorius, 2014). It is proved that AS is a chronic inflammatory disease, in particular, hyperlipidemia, inflammation, and oxidative stress will aggravate it (Zhang et al., 2012). Statins are prominent in treating AS at present, but they have potential adverse reactions and obvious muscle side effects as well as certain adverse reactions to cognitive function, nerve, pancreas, liver function and sexual function (Suguro et al., 2018). Moreover, their treatment cycle is long and expensive, placing a huge economic burden on patients. It turns out that leonurine has a strong protective effect on cardiovascular system, and has a great potential to prevent and treat AS.

Leonurine plays a potential role in AS treatment of by reducing high cholesterol. It is well known that hypercholesterolemia plays a key role in the pathogenesis of AS. Hyperglycemia, high triglyceride, hypercholesterolemia, high and low density lipoprotein can increase the risk of AS. Some studies have shown that the increase of low density lipoprotein (LDL) in plasma promotes the occurrence and development of AS (Ismael et al., 2015). Zhang et al. (2012) established the atherosclerotic model of hypercholesterolemic rabbits to study the preventive potential of leonurine on AS, the effect of leonurine on atherosclerotic plaque formation was observed in hypercholesterolemic rabbits, and with the effect of leonurine on low density lipoprotein. Through the experiment, it was found that high cholesterol diet could promote AS in rabbits, while leonurine treatment can reduce atherosclerotic lesions in experimental rabbits, resulting in the decrease of atherosclerotic plaque and the content of total cholesterol. Similarly, Suguro et al. (2018) established atherosclerotic models in rhesus monkeys, and further explored the potential mechanism of anti‐atherosclerotic effects of leonurine, the results showed that leonurine could reduce the levels of serum total cholesterol (TC), triglyceride (TG) and LDL, which provided evidence for treating atherosclerotic diseases, and it would become a substitute for statins drugs because of the tolerability of leonurine. These research results laid a solid foundation for further clinical trials of leonurine.

Inflammation and oxidative stress were closely related to the pathological process of AS (Kattoor et al., 2017). Oxidative stress caused by excessive reactive oxygen species (ROS) might play an important role in the chronic inflammatory response of AS resulted from hypercholesterolemia. Inflammatory response involves a variety of inflammatory cells, inflammatory cytokines, inflammatory mediators, adhesion molecules, chemokines and growth factors. Inflammation‐related molecules (including ICAM‐1, VCAM‐1, IL‐6, TNF‐α, MCP‐1, iNOS, and MMP‐9) coordinate with oxidative stress to activate atherosclerosis. It has been proved that leonurine has anti‐inflammatory effect, which is expected to become a new way to prevent and treat AS. Leonurine can prevent the early pathogenesis of AS via regulating vascular inflammation. And it could also inhibit the process of AS in a dose‐dependent manner by reducing macrophage infiltration and smooth muscle cell migration, and reducing the expression of some cytokines (IL‐6, TNF‐α), adhesion molecules (VCAM‐1, ICAM‐1) and chemokine (MCP‐1). At the same time, leonurine could also reduce inflammation by inhibiting the production of intracellular ROS, as well as inhibiting the activation of p38 and MAPK pathways, and finally blocking the activation of NF‐κB (Liu, Zhang, et al., 2012). Leonurine was able to down‐regulate the levels of iNOS and COX‐2, and increase the levels of GSH, SOD, CAT, and GPx, which confirmed the antioxidant stress effect of leonurine (Zhang et al., 2012). In a word, although there are many pathogenesis of AS, which can be cured by leonurine.

#### 2.1.2 Treatment of myocardial infarction caused by ischemic myocardial injury

Myocardial infarction (MI), a ischemic heart disease, which is serious and harmful cardiovascular disease to human health. Myocardial ischemia refers to the insufficient blood supply of coronary artery caused by incomplete occlusion of coronary artery blood flow, however, severe persistent ischemia causes serious damage to cardiomyocytes, resulting in MI caused by partial myocardial necrosis (Burke & Virmani, 2007). Oxidative stress and apoptosis have been proved to play a key role in the pathogenesis of MI. Oxidative stress is the common medium of apoptosis, reactive oxygen species (ROS) is directly involved in the occurrence of apoptosis, therefore, hypoxic cardiomyocytes are accompanied by cardiomyocyte apoptosis (Munzel et al., 2017). Studies have shown that apoptosis is one of the causes of cell death during MI, especially near the infarcted myocardium (Fang et al., 2011). Therefore, antioxidative stress and anti‐apoptosis therapy may be effective strategies to reduce MI induced by ischemia (Fang et al., 2011). On the one hand, MI is accompanied by a large number of ROS accumulation, NOX4 is the main source of ROS in injured heart (Kuroda et al., 2010). Leonurine played a significant role in antioxidation to improve cardiac function in patients with MI by inhibiting the expression of NOX4 and blocking the production of ROS (Liu, Pan, et al., 2013).

On the other hand, PI3K/Akt is an important signal pathway mediating survival, growth and apoptosis (Zhang, Wang, et al., 2019). Activation of Akt phosphorylation could promote the survival of diseased cardiomyocytes, while inhibition of Akt activity accelerated hypoxia‐induced cardiomyocyte dysfunction. It has been found that leonurine could reduce the infarct size and improve cardiac recovery in patients with MI by inhibiting apoptosis in infarcted area. The mechanism might be that leonurine regulates apoptosis mediated by PI3K/Akt/GSK3β signal pathway (Xu et al., 2018). Phosphorylated PI3K activated Akt, further inhibited the phosphorylation of glycogen synthase kinase‐3β (GSK3β), the downstream target of Akt, and finally down‐regulated the expression of pro‐apoptotic genes Bax and Caspase3, and up‐regulated the expression of anti‐apoptotic genes Bcl‐2 and Bcl‐xL (Zhang et al., 2015). In addition to its anti‐apoptotic effect, leonurine might also play a role in promoting angiogenesis through PI3K/Akt signal pathway to improve ischemia‐induced infarcted myocardial injury. Hypoxia inducible factor (HIF‐1) and vascular endothelial growth factor (VEFG) play an important role in promoting angiogenesis (Qi et al., 2017). Leonurine promotes the phosphorylation of Akt, further induces the accumulation of downstream signal factor HIF‐1, and increases the expression of VEFG (Liu, Pan, Gong, et al., 2010).

#### 2.1.3 Cardioprotective effect

Hypoxia and apoptosis have great harm to cardiac cells, which can lead to ischemic heart disease, myocardial infarction, and heart failure. Initially, it was found that leonurine could dilate blood vessels and inhibit the contraction of vascular smooth muscle, which may be related to calcium channels (Chen & Kwan, 2001). Liu et al. (2009) found that leonurine could promote the release of calcium ions from sarcoplasmic reticulum, enhance myocardial contractility, up‐regulate the expression of sarcoplasmic reticulum calcium pump and down‐regulate the expression of Ca^2+^, promote calcium ion to return to sarcoplasmic reticulum, block myocardial injury caused by Ca^2+^ overload, and improve cardiac function. Furthermore leonurine could reduce the apoptosis induced by DOX, reduce the formation of malondialdehyde (MDA) and intracellular Ca^2+^ overload, improve the respiratory function of myocardial mitochondria, and has a strong cardioprotective effect in H9c2 cells (Xin et al., 2009). Wang et al. (2019) proved that leonurine could also protect myocarditis induced by lipopolysaccharide (LPS) through antioxidation and anti‐inflammation. And leonurine had an effective cardioprotective effect on myocarditis by inhibiting the activation of NF‐κB pathway and reactive oxygen species (ROS) (Liu, Zhang, et al., 2012). In addition, Liu, Chen, et al. (2009) have proved that leonurine could provide cardioprotective effect, and increase the vitality of cardiomyocytes injured by hypoxia. After hypoxia, leonurine plays a cardioprotective role by up‐regulating the expression of anti‐apoptosis genes Bcl‐2 and Bcl‐xL as well as down‐regulating the expression of pro‐apoptosis genes Fas and Bax. At the same time, leonurine reduces the production of ROS via enhancing the scavenging effect of antioxidants and increasing the activities of antioxidant enzymes SOD and CAT, which leads to the inhibition of oxidative stress.

### 2.2 Treatment of central nervous system diseases

**Figure 3:**
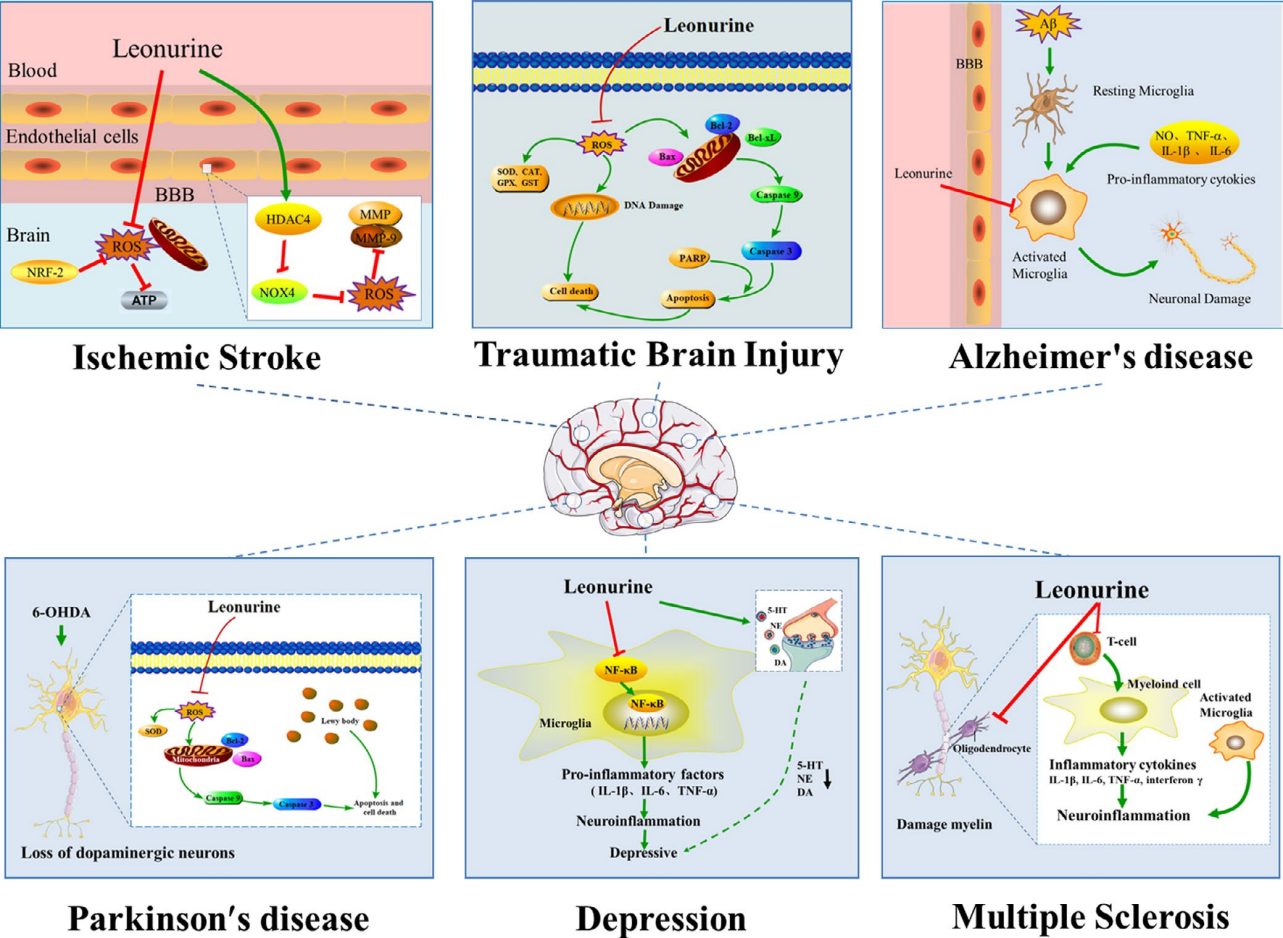
Potential effect of leonurine on central nervous system diseases. It has a strong protective effect on the brain through a wide range of mechanisms. In ischemic stroke, leonurine has a good inhibitory effect on oxidative stress by regulating HDAC4/NOX4/MMP‐9 signal pathway through antioxidant activity. In traumatic brain injury, leonurine can improve brain injury through the mechanism of anti‐apoptosis and antioxidation. In Alzheimer's disease, leonurine can inhibit the overactivation of microglia and reduce the expression of inflammatory cytokines NO, TNF‐α, IL‐1β, and IL‐6, which has obvious anti‐inflammatory effects. In Parkinson's disease, leonurine plays an important role in antioxidant stress, increases the ratio of Bcl‐2/Bax, and has neuroprotective effect. In depression, leonurine alleviates depression by inhibiting the activation of NF‐κB signal pathway and increasing the levels of 5‐HT, NE, and DA. In multiple sclerosis, leonurine reduces inflammation and myelin damage in the central nervous system by inhibiting the recruitment of autoimmune T cells to the central nervous system

In addition to cardiovascular disease, leonurine also had a significant therapeutic effect on central nervous system diseases. In particular, central nervous system diseases such as ischemic stroke, brain injury, Alzheimer′s disease, Parkinson′s disease, depression and multiple sclerosis have become major threats to human lives and health. Many studies have proved that leonurine could ameliorate these diseases. Therefore, leonurine is expected to enter clinical trials as a new drug for the treatment of central nervous system diseases in the future. This section reviews the treatment effects of leonurine on some common central nervous system diseases (Figure 3).

#### 2.2.1 Treatment of ischemic stroke

Stroke, also known as cerebral apoplexy, is one of the main clinical types of cerebrovascular diseases, including ischemic stroke (IS) and hemorrhagic stroke, in which IS is the most common type of stroke, and is known as cerebral infarction (Xu et al., 2017; Zhang, Wang, et al., 2019). IS is caused by inadequate blood supply to the brain for various reasons, which results in cerebral ischemia and hypoxic necrosis (Hao et al., 2020). Leonurine has a good therapeutic effect on IS and may be a very good choice for the treatment of stroke.

Loh et al. (2010) used middle cerebral artery occlusion as a research model to evaluate the therapeutic effect of leonurine on IS. The results showed that the activities of SOD and GPX decreased and the level of MDA increased in IS model rats induced by cerebral artery occlusion. Serious oxidative stress occurred and the activity of free radicals increased will result in more serious damages to the brain. After treatment with leonurine, MDA decreased significantly, while the activities of SOD and Gpx increased, which had a good inhibitory effect on oxidative stress. And the process of antioxidation may be associated with the activation of nuclear factor erythroid 2 related factor 2 (Nrf‐2) (Xie et al., 2019). In addition to antioxidant activity, leonurine can reduce the production of mitochondrial reactive oxygen species or mitochondrial oxidative stress, and inhibit the synthesis of ATP, which has a cytoprotective effect on various hypoxic and ischemic injuries. Furthermore, leonurine can slow down the process of IS by regulating the function of mitochondria (Xie et al., 2019).

In the further study of the mechanism of leonurine's treatment of IS, Zhang et al. (2017) found that leonurine played a key role in reducing the permeability of the blood–brain barrier. When IS occurs, it will damage the blood–brain barrier and cause more serious reactions such as cell rupture, edema and inflammation. The specific mechanism may be that leonurine protects the integrity of the blood–brain barrier by regulating the HDAC4/NOX4/MMP‐9 signal pathway. Histone deacetylase (HDAC4) is involved in the regulation of the integrity of the blood–brain barrier. Leonurine enhances the expression of HDAC4, while HDAC4 inhibits the activity of NADPH oxidase (NOX‐4). Because NF‐κB and reactive oxygen species (ROS) participate in the activation of matrix metalloproteinase‐9 (MMP‐9), the expression of MMP‐9 is also inhibited. To sum up, leonurine can reduce the damages of neurological function and the permeability of blood–brain barrier, so it may be used as a new drug for the treatment of brain IS.

#### 2.2.2 Treatment of traumatic brain injury

Traumatic brain injury (TBI), known as post‐concussive syndrome, refers to brain injury caused by external forces such as falls, blows, falls, attacks, and traffic accidents (Blennow et al., 2016). TBI includes primary and secondary brain injury, so brain injury may also induce other diseases, such as cognitive impairment, motor dysfunction, permanent dysfunction, epilepsy, and hemiplegia, which are the main causes of death and disability (Blennow et al., 2016). After brain injury, it is more vulnerable to suffer oxidative damage, and excessive oxygen free radicals block mitochondrial respiration, which results in mitochondrial swelling and cell death. Oxidative stress promotes apoptosis and inflammation, and brain cells often lead to necrosis or apoptosis due to ischemic or hypoxic tissue injury, and the release of inflammatory factors aggravates brain injury. In previous studies, leonurine has been proved to have a neuroprotective effect on IS, as well as it has been proved that leonurine had a certain therapeutic effect on brain injury (Liu, Zhang, et al., 2012). In the study of Yi, it was found that leonurine could improve brain injury through anti‐apoptosis and antioxidant mechanisms (Yi, 2013). Leonurine increases the activities of SOD, CAT, GPx and GST in brain tissue. At the same time, the expression of pro‐apoptotic proteins Bax and Poly (ADP‐ribose) polymerase (PARP) decreased significantly, while the expression of anti‐apoptotic proteins Bcl‐xL and Caspase‐3 increased significantly. In a word, leonurine can significantly increase the level of endogenous antioxidants, and inhibit apoptosis and inhibit brain cell injury. Due to its excellent therapeutic effect, leonurine can be further developed into a new drug for treating TBI in the future.

#### 2.2.3 Treatment of Alzheimer's disease

Alzheimer's disease (AD), a chronic neurodegenerative disease, often occurs in the elderly, and AD patients ordinary have serious phenomena such as cognitive impairment and memory impairment (Sarlus & Heneka, 2017). Senile plaque formed by the accumulation of amyloid β (Aβ) is a typical feature of AD (Hong et al., 2014). The accumulation of a large number of plaques in the brain will cause memory impairment and cognitive impairment, and further lead to the occurrence of AD. And microglia participates in the production and accumulation of Aβ. Some studies have shown that microglia has neurotoxicity, and its excessive activation is one of the important causes of AD. At the same time, excessive activation of microglia can also promote the release of many inflammatory factors, such as NF‐κB, TNF‐α, IL‐6, etc., which will cause neuroinflammation and more serious harm (Haque et al., 2018). Hong et al. (2014) established a rat model of cognitive impairment induced by Aβ_1‐40_. The results showed that leonurine could inhibit the overactivation of microglia, and had obvious anti‐inflammatory effect by inhibiting the activation of c‐Jun N‐terminal kinase (JNK) and NF‐κB signal pathway in microglia, as well as reducing the expression of inflammatory factors NO, TNF‐α, IL‐1β and IL‐6. Therefore, leonurine can effectively inhibit the excessive activation of microglia, directly or indirectly protect neurons, reduce neuronal death, and improve the cognitive ability of rats with cognitive impairment. At present, there are many drugs treating for AD, but nonsteroidal anti‐inflammatory drugs are associated with gastrointestinal, cardiovascular or nephrotoxic side effects, thus leonurine may be a potential candidate for treating AD.

#### 2.2.4 Treatment of Parkinson′s Disease

Parkinson′s Disease (PD) is a worldwide neurodegenerative dyskinesia disease. Its pathology is characterized by selective loss of dopaminergic neurons in the substantia nigra compacta. In addition, PD is a dopaminergic disorder with accumulation of Lewy body in the remaining nerves. The clinical phenomena such as motor retardation, stiffness, tremor and postural balance disturbance are very common in the middle‐aged and elderly people. Some studies have shown that mitochondrial function, oxidative stress and apoptosis played a key role in the pathogenesis of PD (Poewe et al., 2017). Previous studies have proved that leonurine had the effects of antioxidant stress and anti‐apoptosis, and it could reduce the role of reactive oxygen species in mitochondria. Therefore, it is investigated whether leonurine has a good therapeutic effect on PD. Shi et al. (2011) used the PD model induced by 6‐hydroxydopamine (6‐OHDA) to study the therapeutic effect of leonurine on nerve injury. 6‐OHDA has certain neurotoxicity resulted from oxidative stress, and it can produce too much reactive oxygen species. By up‐regulating the expression of superoxide dismutase (SOD) and reducing reactive oxygen species, leonurine plays a significant role in antioxidant stress, which can reduce the cell death of SH‐SY5Y cells induced by 6‐OHDA and have a neuroprotective effect. Mitochondrial function is involved in the important process of apoptosis, and is a key regulator of cell survival and death. 6‐OHDA‐induced mitochondrial membrane potential losses in the model, but leonurine can exert anti‐apoptotic effects by maintaining mitochondrial function. In addition, the increased Bcl‐2/Bax ratio also serves as a key indicator in the anti‐apoptotic effect. Therefore, leonurine can reduce the abnormal behavior of rats with 6‐OHDA injury, and has neuroprotective effect in vivo and in vitro, which provides a useful reference value for treating PD.

#### 2.2.5 Treatment of depression

Depression is a kind of mental illness with high mortality and morbidity. With the increase of life pressure, more and more people suffer from depression in the world (Otte et al., 2016). Patients with depression often show lack of interest in life, loss of appetite, cognitive defects, and even suicidal thoughts, which not only brings pain to themselves and their families, but also becomes a serious social medical problem (Meng et al., 2019). Previous studies have found that several viewpoints related to the pathogenesis of depression: monoamine neurotransmitter hypothesis, hypothalamus–pituitary–adrenocortical (HPA) axis hyperactivity hypothesis, brain neurotrophin hypothesis, and oxidative stress hypothesis, in which monoamine neurotransmitter hypothesis is considered to be the most reliable viewpoint, has and has become the main target of clinical antidepressants. The contents of 5‐hydroxytryptamine (5‐HT), norepinephrine (NE) and dopamine (DA) in the brain decrease for patients with depression. Neuroinflammation also plays an important role in the pathological process of depression, and many cytokines and inflammatory factors are also involved in this process, such as, IL‐1β and IL‐6. As a key transcription factor in the production of depression, NF‐κB can regulate inflammatory response. Jia et al. (2017) explored the therapeutic effect of leonurine on severe depression by establishing a chronic mild stress mouse model of depression (CMS). The research results showed that leonurine could reduce the depressive behavior of CMS mice, restore the level of monoamine neurotransmitters and improve the pathological damage of hippocampus. In addition, leonurine alleviates CMS‐induced neuroinflammation by inhibiting the activation of NF‐κB signaling pathway, and it increases the levels of 5‐HT, NE and DA by inhibiting inflammatory response. Besides, leonurine has an anti‐inflammatory effect by inhibiting the overactivation of microglia. In the latest study, Meng et al. (2019) reported that leonurine could promote neurite growth and neurotrophic activity by regulating glucocorticoid receptor/glucocorticoid‐induced kinase 1 (GR/SGK1) signal pathway, and had antidepressant effect in corticosterone (CORT)‐induced PC12 cell depression model. At present, there are some classical antidepressants such as fluoxetine in the market, but most of them have some defects, such as weak antidepressant efficacy, narrow depression spectrum, slow onset, large adverse reactions and short action time. Therefore, so far the development of new antidepressant drugs still is a difficult task. Leonurine has antidepressant effects by improving monoamine neurotransmitters and inhibiting nerve inflammation, it may be one of the effective strategies for the treatment of depression in the future.

#### 2.2.6 Treatment of multiple sclerosis

Multiple sclerosis (MS) is an ordinary autoimmune disease of the central nervous system with the most common chronic inflammation, demyelination, and neurodegeneration, which usually occurs in young people, and women are more likely to get sick than men (Filippi et al., 2018). Patients often experience limb weakness and neurological impairment, and even endanger lives. Therefore, inhibiting the neuroinflammatory response and promoting myelin sheath regeneration are effective strategies for the treatment of MS. It is well known that demyelination caused by specific T cell response mediates the production of MS and EAE models. T cells participate in immune response, causing the production of inflammatory cytokines IL‐1β, IL‐6, IL‐17, TNF‐α and interferon γ, as well as severe neuroinflammatory responses, which in turn lead to the damage of myelin sheath and axon. Oligodendrocytes play a crucial role in promoting the regeneration of myelin. Jin et al. (2019) established a model of experimental autoimmune encephalomyelitis (EAE) induced by myelin peptide MOG_35‐55_ and investigated the therapeutic effect of leonurine on MS. The results showed that after treatment with leonurine, MOG_35‐55_‐induced multiple sclerosis in EAE model mice was alleviated, which was characterized by less inflammatory cell infiltration and demyelinating plaque, indicating that the inflammation and myelin injury of the central nervous system were significantly alleviated. The inhibitory effect of leonurine on inflammation may be achieved through inhibiting the recruitment of autoimmune T cells to the central nervous system. In addition, leonurine not only reduced the myelin damage, but also promoted the formation of remyelination by promoting the differentiation of oligodendrocyte. Therefore, leonurine has a potential therapeutic effect on MS.

## 3 STRUCTURE AND STRUCTURE–ACTIVITY RELATIONSHIP

Ever since leonurine was isolated in 1930 and its chemical structure was determined in 1969, scientists have never stopped exploring it (Yeung et al., 1977). Its molecular formula is C_14_H_21_O_5_N_3_ and the structure of leonurine is characterized by a guanidino, an n‐butyl and a syringate. Unlike other alkaloids, leonurine has a unique guanidino, as well as a hydroxyl group on the benzene ring. The above review shows that leonurine has significant therapeutic effects on cardiovascular system and central nervous system diseases, but the high polarity of guanidine and hydroxyl groups brings some difficulties to clinical application, including low bioavailability, weak transmembrane ability and poor fat solubility. Some studies have shown that guanidine is still the key group for the binding of leonurine to the site of action. Therefore, many pharmaceutical chemists have tried to modify its structure by using bioisosterism and drug combination principles, as well as codrug or mutual prodrug. For example, combining aspirin with phenolic hydroxyl groups could enhance the pharmacological activity of leonurine, reduce toxicity and side effects, and improve its bioavailability and pharmacokinetic properties. It could significantly improve the cardioprotective effect of leonurine by increasing the activity of antioxidant enzymes, reducing the level of MDA and inhibiting inflammatory mediators (Gao et al., 2016). In addition, Liu et al. (2011) found that the new conjugate “leonurine‐cysteine” could increase the activity of SOD and CAT and reduce the level of MDA and ROS. By combining cysteine and leonurine, it can exhibit a stronger protective effect in myocardium. In addition to enhancing the cardioprotective effects, structural modifications can also promote the potential therapeutic effects on brain diseases. Que et al. (2013) replaced guanidino with mercaptoethylleonurine (MEL), to synthesize a new leonurine analog, which showed a neuroprotection via the effect of anti‐apoptosis by up‐regulating the level of Bcl‐2 and inhibiting the expression of Bax.

**Figure 4:**
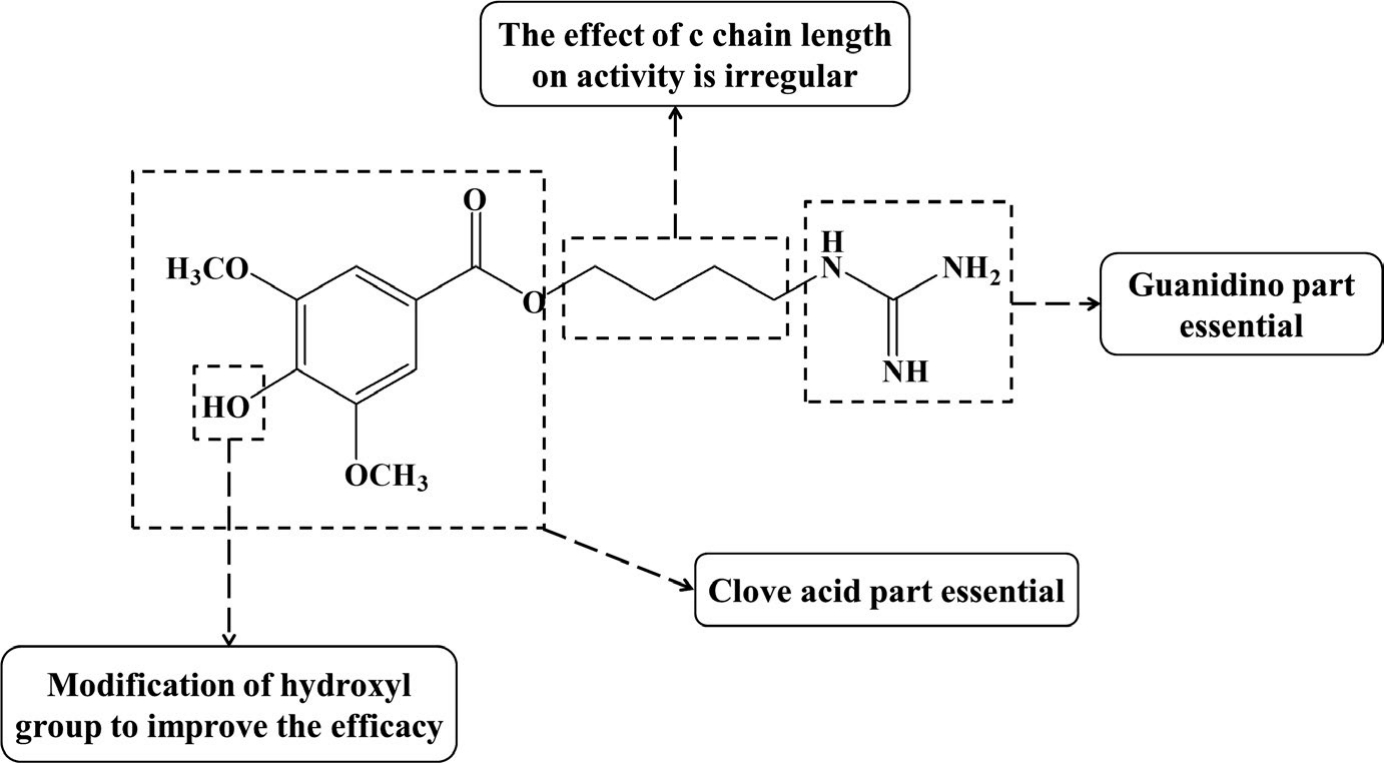
Structure–activity relationship of leonurine

To sum up, the structural modification of leonurine mainly focus on the combination of aspirin, cysteine, SPRC and other groups or compounds with hydroxyl groups to enhance the pharmacological action of leonurine and improve the bioavailability. At the same time, it is found that although guanidine groups are protected by N, N′‐Boc‐methyl‐thiourea, eventually they are removed during the synthesis (Luo & Gu, 2012). Surprisingly, these new compounds have better cardioprotective and central nervous system protective effects. So, we think that the design and structural modification of leonurine may be expected to develop new potential drugs in the future (Figure 4).

## 4 CONCLUSION AND PROSPECT

This article reviews the pharmacological effects of a hot potential drug leonurine on cardiovascular system and central nervous system diseases, such as ant‐oxidation, cardiovascular protection, neuroprotection, and anti‐apoptosis. Its clinical effects are investigating or are prepared to study in further trials. Its structure modification may be an effective way to reduce side effects, strengthen efficacy, and improve bioavailability. Although leonurine shows good pharmacological or therapeutic effects, the potential biological targets still are unclear. Leonurine has attracted worldwide attention as a potential new drug with obvious efficacy in reducing blood fat and treating cerebral stroke. We believe that in the near future, leonurine is very likely to become one of the five original Class 1 new drugs following the results of artemisinin, which will benefit all human beings.

## CONFLICT OF INTEREST

The authors declare no conflict of interest.

## AUTHOR CONTRIBUTIONS

Lu Huang searched the literature and drafted the manuscript. Ding‐Qiao Xu and Yu‐Ping Tang conceived and designed the review. Yan‐Yan Chen and Shi‐Jun Yue examined the literature and made the figures. Ding‐Qiao Xu and Yu‐Ping Tang made a critical revision of the review. All authors contributed to the article and approved the submitted version.

## FUNDING INFORMATION

National Key R&D Program of China (2019YFC1711000), the National Natural Science Foundation of China (81773882, 81974522), Key Research and Development Program of Shaanxi Province (2019ZDLSF04‐05), and Subject Innovation Team of Shaanxi University of Chinese Medicine (2019‐YL10).

## Data Availability

Data sharing is not applicable to this article as no new data were created or analyzed in this study.
